# Machine learning for suicide risk prediction in children and adolescents with electronic health records

**DOI:** 10.1038/s41398-020-01100-0

**Published:** 2020-11-26

**Authors:** Chang Su, Robert Aseltine, Riddhi Doshi, Kun Chen, Steven C. Rogers, Fei Wang

**Affiliations:** 1 Department of Population Health Sciences, Weill Cornell Medicine, New York, NY USA; 2 Division of Behavioral Sciences and Community Health, UConn Health, Farmington, CT USA; 3 Center for Population Health, UConn Health, Farmington, CT USA; 4 Department of Statistics, University of Connecticut, Storrs, CT USA; 5 Connecticut Children’s Medical Center, Hartford, CT USA

**Keywords:** Psychiatric disorders, Diseases

## Abstract

Accurate prediction of suicide risk among children and adolescents within an actionable time frame is an important but challenging task. Very few studies have comprehensively considered the clinical risk factors available to produce quantifiable risk scores for estimation of short- and long-term suicide risk for pediatric population. In this paper, we built machine learning models for predicting suicidal behavior among children and adolescents based on their longitudinal clinical records, and determining short- and long-term risk factors. This retrospective study used deidentified structured electronic health records (EHR) from the Connecticut Children’s Medical Center covering the period from 1 October 2011 to 30 September 2016. Clinical records of 41,721 young patients (10–18 years old) were included for analysis. Candidate predictors included demographics, diagnosis, laboratory tests, and medications. Different prediction windows ranging from 0 to 365 days were adopted. For each prediction window, candidate predictors were first screened by univariate statistical tests, and then a predictive model was built via a sequential forward feature selection procedure. We grouped the selected predictors and estimated their contributions to risk prediction at different prediction window lengths. The developed predictive models predicted suicidal behavior across all prediction windows with AUCs varying from 0.81 to 0.86. For all prediction windows, the models detected 53–62% of suicide-positive subjects with 90% specificity. The models performed better with shorter prediction windows and predictor importance varied across prediction windows, illustrating short- and long-term risks. Our findings demonstrated that routinely collected EHRs can be used to create accurate predictive models for suicide risk among children and adolescents.

## Introduction

Suicide among children and adolescents is one of the most critical public health concerns^1–5^. As the second leading cause of death among children, adolescents and young adults between ages 10–24 years, suicide claims over 6000 young lives every year in the US alone^6^. There has been an alarming increase in suicide rates among those 10–24 years of age^7^. Compared to 2000, suicide rates were 2–3 times higher in 2016 for this population^8^. The burden of suicide attempts is many folds higher than suicide deaths. In 2017, about 1 in 6 adolescents and young adults seriously considered attempting suicide and 1 in 13 attempted suicide^9^. Every year approximately 700,000 adolescents seek healthcare after an attempted suicide^10^.

In clinical practice, predicting the risk of suicide accurately within an actionable time frame is critical^11–14^. However, most of the clinical risk assessment tools available to clinicians are not sufficiently accurate to identify high-risk patients^14–17^. While the Columbia Suicide Severity rating scale presents an effective alternative for older clinical assessment tools, it may not be possible to screen every patient with this tool during the clinical encounter. Hence, it is evident that clinical practitioners need more than just clinical assessment tools to identify patients at risk of suicide. Recent efforts to apply machine learning with electronic health record (EHR) data to predict suicide risk in adult populations^18–23^ have not only confirmed the importance of prominent risk factors for suicidal behavior identified in prior research^24–27^ but also identified other characteristics leading to improved accuracy in suicide prediction compared to previous efforts^19,28,29^. However, there is scarcity of advanced risk prediction models that can be applied in clinical practice to the general population of children, adolescents, and young adults. Walsh et al.^30^ constructed a predictive model for adolescents who required no in-person assessment using EHR data with good prediction performance in a limited population in the Southern US. However, there is a need for a more comprehensive risk predictive models that consider a wider range of clinical factors beyond mental health comorbidities and medications and generate quantifiable risk scores that can be applied to determine the patient’s risk level for suicidal behavior.

In this retrospective study, we examined a range of demographic, diagnostic, laboratory, and medication-related factors derived from EHRs to identify significant predictors for suicidal behavior among children and adolescents receiving inpatient and outpatient care at a major children’s hospital to compare and contrast predictors across different time windows to examine the potential for variability in the factors associated with short- and longer-term risk. This is one of the few analyses in the suicide risk modeling literature to explicitly differentiate models predicting near term vs. longer term risk.

## Methods

### Ethics statement

Data analyzed in this study were deidentified by removing protected personal health information. Our study was approved by the University of Connecticut Health Center Institutional Review Board and Weill Cornell Medical College Institutional Review Board.

### Cohort

**Fig. 1:**
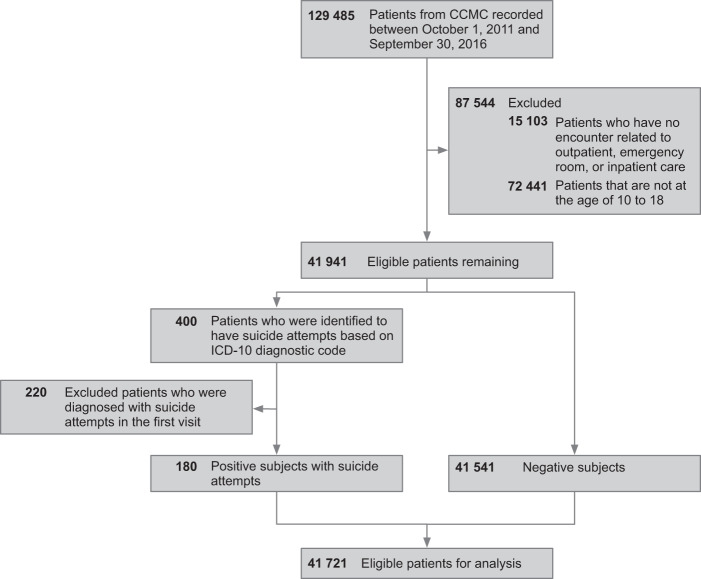
Study flow outlining exclusion criteria. CCMC Connecticut Children’s Medical Center, ICD-10 International Classification of Diseases, Tenth Revision.

We analyzed data from the Connecticut Children’s Medical Center (CCMC) EHR database, which contains information on 641,708 visits among 129,485 patients from 1 October 2011, to 30 September 2016. We included in the analysis all encounters related to outpatient, emergency room, or inpatient care among patients between 10 and 18 years of age at the time of the first suicide diagnosis (for whom has the suicide attempts) or the last visit (for whom has no suicide attempt). Patients who have no encounter related to outpatient, emergency room, or inpatient care were considered as missing longitudinal data and hence were excluded for analysis. In addition, patients whose first recorded visits due to suicide attempts were excluded since the lack of longitudinal information. To identify suicide attempts, we used an algorithm including both the external cause of injury codes of International Classification of Diseases, Ninth Revision (ICD-9) and other ICD-9 code combinations that are indicative of suicidal behaviors (as shown in Supplementary Table 1)^31,32^. Since our data included diagnostic codes in ICD-10 format, we converted the ICD-9 codes from the algorithm to ICD-10, using a public toolkit, AHRQ MapIT^33^. Figure 1 summarizes the data screening process to define the study population and suicide-positive and -negative groups for this study.

### Measures and outcomes

To gain insight on both short- and long-term suicide risk and how the risk factors evolve through different periods of observation, we developed a series of models predicting the risk of future suicide attempts with varying prediction windows. We constructed models for prediction windows, including 0, 7, 14, 30, 60, 90, 180, 270, and 365 days. The model for each prediction window only uses data equal to or more distant in time than the length of the window. For example, the model for a prediction window of 60 days aims to predict the occurrence of a future suicide attempt that happens 60 days or longer following the encounter. Consequently, the model is trained using patient’s data that are captured at least 60 days prior to their end points, where the end point for a suicide-positive subject is defined as the time of his/her first suicide attempt, and the end point of a negative subject is defined as the time of his/her last recorded clinical visit. The model trained with the 0 day prediction window is expected to predict the suicide risk any time after the latest encounter. See Supplementary Fig. 1 and Table 2 for an illustration.

We utilized a broad range of variables as candidate predictors, including patients’ demographic characteristics, diagnosis codes, prescribed medications, and laboratory test data. Demographic characteristics include age, sex, race, and ethnicity. All diagnosis information was presented in ICD-10 format. For medication data, we used a publicly available toolkit MedEx^34^ based on RxNorm^35^ to standardize the prescribed medications. Laboratory data include the name of the laboratory test and its results. The predictors were constructed as binary variables, each of which indicates the absence or presence of a particular factor related to diagnoses, medications, or laboratory tests. We also included all the two-way interactions among these binary variables as candidate predictors.

### Predictor screening

Predictor screening was performed based on testing the marginal correlation of each predictor and the occurrence of suicide attempts. Specifically, each predictor and the occurrence of suicide attempts produced a 2 × 2 contingency table. If all the cells were bigger than 5, then a Chi-square test was used to test the association, else Fisher’s exact test was used. To adjust for multiple testing, we implemented *p* value correction using the Benjamini–Hochberg procedure^36^ to control the false discovery rate (FDR) at 10%. As such, all the predictors with an adjusted *p* value smaller than 0.1 were used as candidate predictors to build the predictive models. We also applied the marginal screening procedure on the interaction variables (see Supplementary Appendix 1 for more details).

### Model development and predictor selection

For each prediction window, we randomly divided the data into 90% training and 10% testing sets. As shown in Supplementary Fig. 2, we first performed the predictor screening to select the informative candidate predictors. Next, we developed the predictive model using the training set. A logistic regression classifier was built via a sequential forward selection procedure^37^ for selecting predictors to minimize the prediction error. The use of the sequential forward selection procedure allows us to watch the order in which predictors are added, and hence can provide valuable information about the quality of the candidate predictors. We concluded the selection procedure when predictive performance, as judged by a fivefold cross-validation, reached the peak. We repeated all the above procedures 10 times to validate the effectiveness of our predictive model and identify the predictors with the strongest association with suicide attempts. The level of importance of each predictor was measured by its frequency of being selected among the 10 predictive models, based on which the final set of predictors was determined. Supplementary Appendix 2 provides more details about the method for constructing predictive model for each prediction window.

### Model evaluation

In order to estimate the proposed predictive models, we implemented logistic regression with L1 regularization for comparisons. The L1 logistic regression models were trained based on candidate predictors passed the univariate screening. To evaluate the predictive performance of the models, we examined out-of-sample performance metrics, including area under the receiver operator characteristic curve (AUC), sensitivity, specificity, and positive predictive value (PPV). AUC is a broad metric of discrimination performance in the machine learning community that ranges from 0.5 (random guessing) to 1.0 (perfect prediction). Since our dataset is highly imbalanced, i.e., 180 suicide-positive subjects vs. 41,541 suicide-negative subjects, we calculated sensitivities when setting specificities to 90% and 95%, respectively. We also calculated PPV, which is the probability that predicted high-risk patients have actual suicide attempts. For each prediction window, performance metrics were calculated based on prediction results over the testing set.

To further evaluate the usefulness of our predictive models, we grouped the selected predictors into seven categories: demographics, depression-related factors (including both diagnoses and medications), other mental health-related factors, routine tests, drug tests, pregnancy-related factors (among females), and other factors. We implemented logistic regression analysis on female and male cohorts, respectively, to estimate the contribution coefficient for each category.

## Results

Following application of our inclusion and exclusion criteria, 180 (0.43%) patients with suicide attempts were labeled as positive subjects, and 41,541 patients without suicide attempts were labeled as negative subjects. Table 1 illustrates the demographic characteristics of the study population.

**Table 1:** Demographics of 41,941 patients of the study cohort.

Variable	*n* (%)	
	Suicide-positive subjects (*n* = 180)	Suicide-negative subjects (*n* = 41,541)
Male sex	37 (20.6)	20,931 (50.4)
Age		
10–12 years old	10 (5.6)	14,064 (33.9)
13–15 years old	81 (45.0)	15,072 (36.3)
16–18 years old	89 (49.4)	12,405 (29.8)
Race		
White or Caucasian	101 (56.1)	18,485 (44.5)
Black or African American	25 (13.9)	5515 (13.3)
Asian	2 (1.1)	611 (1.5)
Other	52 (28.9)	16,930 (40.7)
Ethnicity		
Hispanic or Latino	51 (28.3)	10,951 (26.4)
Not Hispanic or Latino	126 (70.0)	26,688 (64.2)
Unknown or patient refused	3 (1.7)	3902 (9.4)

### Model performance

Patients with no clinical records before the prediction window were excluded for training the model. Hence as the prediction window increases, the number of patients eligible for analysis decrease (see Table 2). Though we analyzed the effects of two-way interactions among the predictors on the risk of suicidal behavior, we ultimately excluded them for building predictive models due to poor predictive performance when they were included. This was mainly due to the general sparseness of the data which was exacerbated by noise introduced by the interaction terms.

**Table 2:** Overall performance of the predictive models.

Prediction window (days)	No. (%) of positive subjects/No. (%) of negative subjects	Model	Average no. of selected predictors (95% CI)	AUC (95% CI)	Sensitivity (SD)		Positive predictive value (SD)	
					90% Specificity	95% Specificity	90% Specificity	95% Specificity
0	180 (0.43)/41,541 (99.57)	Proposed model	18.8 (17.0, 20.6)	**0.84 (0.82–0.87)**	**0.65 (0.06)**	**0.45 (0.07)**	**0.03 (0.00)**	0.04 (0.01)
		Logistic regression L1	–	0.81 (0.77–0.85)	0.52 (0.15)	0.40 (0.14)	0.02 (0.01)	0.04 (0.01)
7	177 (0.91)/19,351 (99.09)	Proposed model	16.2 (15.0, 17.4)	**0.86 (0.84–0.88)**	**0.60 (0.10)**	**0.43 (0.11)**	**0.06 (0.01)**	**0.08 (0.01)**
		Logistic regression L1	–	0.82 (0.78–0.85)	0.56 (0.13)	0.39 (0.14)	0.05 (0.01)	0.07 (0.02)
14	175 (0.93)/18,844 (99.07)	Proposed model	17.4 (15.3, 19.5)	0.85 (0.83–0.87)	0.58 (0.07)	0.45 (0.08)	0.06 (0.01)	0.08 (0.03)
		Logistic regression L1	–	0.86 (0.83–0.89)	0.63 (0.11)	0.46 (0.13)	0.06 (0.01)	0.08 (0.02)
30	167 (0.95)/17,620 (99.05)	Proposed model	16.2 (14.7, 17.7)	**0.86 (0.85–0.88)**	0.60 (0.09)	**0.50 (0.09)**	0.05 (0.01)	**0.09 (0.02)**
		Logistic Regression L1	–	0.83 (0.80–0.86)	0.62 (0.10)	0.47 (0.12)	0.06 (0.01)	0.08 (0.01)
60	149 (0.93)/16.002 (99.07)	Proposed model	15.6 (13.4, 17.8)	**0.85 (0.83–0.88)**	**0.57 (0.06)**	**0.44 (0.10)**	**0.06 (0.01)**	0.07 (0.02)
		Logistic regression L1	–	0.82 (0.78–0.86)	0.56 (0.13)	0.41 (0.12)	0.05 (0.01)	0.07 (0.03)
90	139 (0.93)/14,925 (99.07)	Proposed model	14.9 (13.2, 16.6)	**0.85 (0.83–0.87)**	**0.61 (0.07)**	0.40 (0.09)	**0.06 (0.01)**	0.07 (0.02)
		Logistic regression L1	–	0.81 (0.78–0.85)	0.58 (0.14)	0.40 (0.15)	0.05 (0.02)	0.07 (0.03)
180	114 (0.92)/12,452 (99.08)	Proposed model	14.4 (12.8, 16.0)	**0.86 (0.83–0.89)**	**0.60 (0.10)**	**0.47 (0.17)**	0.05 (0.01)	**0.08 (0.03)**
		Logistic Regression L1	–	0.85 (0.82–0.88)	0.57 (0.10)	0.42 (0.16)	0.05 (0.01)	0.07 (0.02)
270	83 (0.80)/10,337 (99.20)	Proposed model	11.4 (9.8, 13.0)	**0.86 (0.83–0.89)**	**0.64 (0.15)**	**0.51 (0.17)**	0.05 (0.02)	**0.08 (0.02)**
		Logistic Regression L1	–	0.84 (0.80–0.87)	0.51 (0.09)	0.44 (0.12)	0.04 (0.01)	0.07 (0.02)
365	60 (0.72)/8306 (99.28)	Proposed model	13.0 (11.9, 14.1)	0.81 (0.78–0.85)	0.53 (0.21)	0.38 (0.19)	0.04 (0.02)	0.05 (0.02)
		Logistic regression L1	–	0.81 (0.77–0.85)	0.54 (0.19)	0.36 (0.18)	0.04 (0.02)	0.05 (0.02)

*AUC* area under the receiver operator characteristic curve, *CI* confidence interval, *SD* standard deviation.

The bolded value indicates that our proposed model achieved a better performance than that of the compared model with a specific prediction window.

Statistics summarizing the ability of our models to predict the suicide risk, including receiver operating characteristic (ROC) curves, AUC, sensitivity at predefined specificity levels, and PPV, are presented in Table 2, and Supplementary Figs. 3 and 4. The proposed models predicted suicidal behavior with an overall AUC > 0.80 across all prediction time windows. The model performed similarly in terms of AUC for 0- to 270-day prediction windows (AUC = 0.84–0.86). The predictive performance declines for the one-year prediction window (AUC = 0.81, 95% confidence interval [CI] 0.76–0.86) since fewer patients had clinical records 1 year before the observation point. For all prediction windows, the model detected 53–62% of suicide cases with 90% specificity. Consistent with the low prevalence (from 0.43 to 0.95%) of suicidal behavior in the studied cohort, the PPVs across all prediction windows ranged from 3 to 6% for 90% specificity, and from 4 to 8% for 95% specificity (see Table 2). Overall, predictive performances of the proposed models were higher than those of the baseline L1 penalized logistic regression models (see Table 2 and Supplementary Fig. 4).

### Predictor importance

**Fig. 2:**
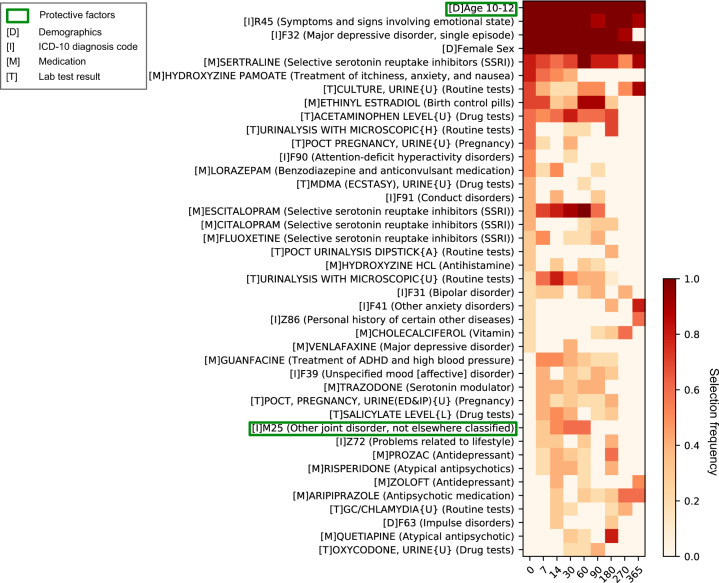
Predictor importance over all prediction windows. Importance of predictors of which summation of frequencies over all prediction windows is no less than 0.5. Predictor importance is measured by frequency that specific predictor is enrolled by the sequential forward selection procedure. Each predictor is shown with its associated type in square brackets embedded as D demographics, I International Classification of Diseases, Tenth Revision (ICD-10) diagnostic codes, M medication, T Lab Test. An asterisk concatenating two variables indicates interaction predictor. In addition, each Lab Test is shown with its associated result in braces embedded as U unspecified, H high, A abnormal.

Figure 2 depicts predictor importance, as measured by the frequency a specific predictor was enrolled by the sequential forward selection procedure. Characteristics of predictors are listed in Supplementary Tables 3–11. The importance of predictors varied across the prediction windows. ICD-10 code R45 (symptoms and signs involving emotional state) and F32 (depressive episode), and gender are the most common risk factors across all prediction windows. Age is also a significant factor, with patients between 10 and 12 years old less likely to attempt suicide than older patients. In addition, antidepressant medications, including sertraline and escitalopram, and urine culture tests are risk factors that show relatively high importance across most prediction windows.

**Fig. 3:**
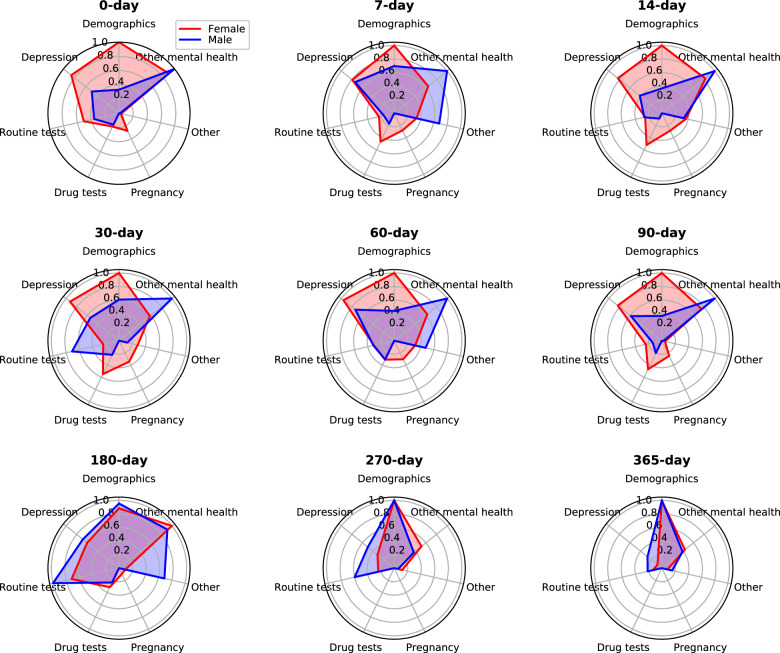
Categorized predictor contribution over all prediction windows. The selected predictors were grouped into seven categories: demographics, depression-related factors (including both diagnoses and medications), other mental health-related factors, routine tests, drug tests, pregnancy-related factors (among females), and other factors. For each prediction window, the value of each predictor category was the normalized cumulative predictor contribution coefficient derived by logistic regression analysis on female patients (red) or male patients (blue), respectively.

To aid in the interpretation of factors having different impacts across short- and longer-term time windows, we grouped the selected predictors into seven categories and calculated the contribution coefficient for each category in identifying suicide risk among both female and male patients (see Fig. 3). Details of each predictor category are listed in Supplementary Table 12. The radar charts presented in Fig. 3 show that, in general, demographics, depression-related factors, and other mental health-related factors were important predictors across all prediction windows. However, as the prediction window lengthens to greater than 180 days several diagnostic factors and laboratory tests are much less useful in predicting suicide risk. In particular, when the prediction window is larger than 270 days, the effects of depression-related and other mental health-related factors became much smaller. For female patients, the effects of female-specific predictors, i.e., pregnancy-related factors, vanish when prediction window is larger than 180 days. Supplementary Figure 5 reveals that the information available for estimating suicide risk, as reflected in the percent of patients in each predictor category having a particular characteristic, declines substantially over time.

## Discussion

Accurate risk prediction plays an important role in the intervention of suicide attempts among children and adolescents. The traditional clinical risk assessment tools have been demonstrated to be not sufficiently accurate to identify high-risk patients^14–17^. Recently machine learning approaches have been applied to EHR data for the prediction of suicide risk in either adult populations^18–22^ or children and adolescent population^30^. Although the previous studies have archived advanced predictive performances compared to the traditional methods, there remains a need for a more comprehensive risk prediction models that utilize clinical data available to produce quantifiable risk scores that can be applied to estimate the patient’s risk level.

In this analysis we have shown that a combination of patient demographic characteristics, diagnoses, procedures, medications, and laboratory tests can be used to construct accurate machine learning models predicting the risk of suicide attempts among pediatric patients receiving inpatient and outpatient care in a children’s medical center. This distinguishes our analysis from virtually all other efforts at suicide risk prediction among both children and adolescents, which have typically had risk horizons of several years to maximize the cases available for analysis^11^. Notably, our models demonstrated good performance over very short time windows, indicating that the detection of short-term risk of suicidal behavior in this population is attainable. In addition, the proposed models accounted for improvements in both short- and long-term prediction, compared to the traditional logistic regression with L1 regularization. The PPVs observed from the proposed models constitute a 5- to 10-fold improvement in suicide attempt prediction compared to the base rate (see Supplementary Table 13), which is superior to that reported in a similar study by Barak-Corren et al.^19^.

Another major finding in this analysis (see Supplementary Figs. 3 and 4, and Table 2) is that time matters how well the models perform. The models performed well in time windows ranging between 7 and 180 days, with declining performance observed 9 months and 1 year prior to the attempt. While this is likely due to the fact that, major identified risk factors, such as depression^24,38,39^, other mental disorders^27,40–42^, and substance abuse^25,43–45^ are proximate risks for suicidal behavior; on the other hand, it is also due to the volume and content of the information available in varying time windows. Supplementary Figure 5 shows that percent of patients whose medical records contained evidence of the identified risk factors falls precipitously over time, indicating that there is a dearth of information with which to construct an accurate suicide risk model among pediatric patients as the time from the previous medical encounter increases beyond 6 months.

In addition, time also matters the attendant risk factors (see Figs. 2 and 3), i.e., contributions of the identified predictors to risk detection vary across prediction windows. In particular, as shown in Figs. 2 and 3, except for demographics, depression-, and other mental disorder-related factors, most predictors shift their importance in risk prediction across prediction windows. This validated the previous finding that majority of the risk factors don’t continuously contribute to the suicide risk^30,46^. Even though identification of the concrete role of an individual risk factor over time is difficult, we did detect predictor importance pattern reflecting short- and long-term risk of suicide behavior. Our produced risk factor panels, as shown in Fig. 3, leads to the potential of assisting the clinical practitioners in assessing risk levels of the patients. First, female’s short-term risk factors (0- to 90-day prediction windows) come from a broader spectrum, as with a higher area within the curve of each radar chart. The important short-term risk factors of female include demographics, depression-, and other mental disorder-related factors. In contrast, the most important risk factors of male are mental disorder-related factors. The findings suggest that, when estimating short-term suicide risk, risk factors of female and male populations could be emphasized differently. In addition, for both female and male, impacts of the non-demographic risk factors for long-term risk estimation (270- and 365-day prediction windows) decrease due to the dearth of information (Supplementary Fig. 5). Besides, depression- and other mental disorder-related factors are also important in predicting long-term risk.

### Strengths and limitations

This study provides a number of critical insights to inform clinical practice. First, we have shown that information that is routinely collected in clinical encounters and maintained in structured clinical records can be used to create accurate predictive models of the risk of suicidal behavior among children and adolescents. Nothing drastically novel was observed among the factors emerging as significant predictors of suicide risk, which is a *good thing*: it means that the information needed to identify at risk patients is readily available and just requires a mechanism to incorporate it into clinical care. Second, not only do we find that short-term risk of suicidal behavior can be detected, but that longer periods between clinical encounters results in less accurate prediction of suicide risk. This indicates that high-risk patients, whether identified through risk algorithms or by clinical history (e.g., a prior attempt), would benefit from ongoing clinical monitoring.

The present study has several limitations. First, the data are restricted to a single clinical setting with a limited number of suicidal events, potentially limiting both its power and generalizability. Among the 41,721 patients eligible for analysis, only 180 (0.43%) are cases with records of suicide attempt. We did not introduce specific strategies to address the positive-negative imbalance of suicide attempt. One possible consequence of this limitation is that certain risk factors, which are associated with suicide risk but appear infrequently in this patient population, would not be identified. This highlights the need of methods addressing imbalanced clinical data analysis^47,48^. Second, data for this analysis were collected from a single institution, i.e., CCMC EHR database. This may lead to the possibility that patients identified as negative subjects due to an absence of suicide records actually have been treated for suicide attempts at other institutions. In addition, drawing training and testing data from the same dataset also downgrades the power and generalizability of our models. Third, mining the text of clinical notes has been demonstrated to enrich the predictive models of suicide attempts^21,22,49^, while in this analysis we did not have access to patients’ clinical notes. Future work may incorporate clinical notes to combine with structured EHR data to enhance our predictive models, but it should be noted that de-identification in clinical text is more complex than that in the structured EHR data^50,51^ and hence needs more attention. Finally, among the 41,721 eligible patients, 19,941 (47.80%) received health insurance through Medicaid, which is slightly higher than the national average for Medicaid coverage among children (38%)^52^. This may lead to bias and potentially limit the generalizability of our findings for commercially insured patients.

## Conclusions

Our study demonstrated the feasibility of creating predictive models of suicide risk of children and adolescents by using demographics, comorbidity diagnosis codes, laboratory test results, and medications from clinical records. Such models showed good predictive performances for estimation of short-term and long-term risks and identified significant predictors which may assist in clinical practices.

## Supplementary information

Supplemental Materials
