# Supplementary material for: Machine learning for suicide risk prediction in children and adolescents with electronic health records
Source: Transl Psychiatry. 2020 Nov 26;10:413. doi: 10.1038/s41398-020-01100-0 (PMC7693189; doi:10.1038/s41398-020-01100-0)
Supplement: Supplementary file 1 — Supplemental Materials [file 41398_2020_1100_MOESM1_ESM.docx]

Supplementary Online Content

### **Appendix 1**. Marginal Screening for Interaction Variables.

### **Appendix 2**. Details of Model Development and Predictor Selection.

### **Appendix 3**. Calculation of Contribution Coefficient.

### **Table 1**. Identification of Suicide-related Admissions using ICD-9 Codes.

**Table 2**. Longitudinal characteristics of the study population.

### **Table 3**. Top 30 Predictors of 0-day Prediction Window.

### **Table 4**. Top 30 Predictors of 7-day Prediction Window.

### **Table 5**. Top 30 Predictors of 14-day Prediction Window.

### **Table 6**. Top 30 Predictors of 30-day Prediction Window.

### **Table 7**. Top 30 Predictors of 60-day Prediction Window.

### **Table 8**. Top 30 Predictors of 90-day Prediction Window.

### **Table 9**. Top 30 Predictors of 180-day Prediction Window.

### **Table 10**. Top 20 Predictors of 270-day Prediction Window.

### **Table 11**. Top 20 Predictors of 365-day Prediction Window.

### **Table 12**. Details of Selected Predictors.

### **Table 13**. Model Improvements across Prediction Windows.

### **Figure 1**. Illustration of Prediction Window.

### **Figure 2**. Illustration of Study Flow.

### **Figure 3**. Receiver Operating Characteristic (ROC) Curves for the Predictive Model over Prediction Windows. The ROC curves illustrate the overall predictive performance of the model across test subjects.

### **Figure 4**. AUC Curve across Prediction Windows. This figure plots prediction performance in terms of AUC with 95% confidence interval cross all prediction windows.

### **Figure 5**. Percent of Patients with Specific Risk Factor.

This supplementary material has been provided by the authors to give readers additional in-

formation about their work.

# Appendix 1. Marginal screening for interaction variables

Let denote by A×B the interaction variable of individual variables A and B. By univariate analysis, the contribution of the interaction variable A×B is usually affected by that of individual variables A and B. To address this, we applied the logistic regression analysis on A×B, A, B, and confounders including age and sex. The p-values were corrected by FDR correction. The interactions that do not reach the significance criterion (corrected p-value < 0.1) were excluded for building predictive model.

# Appendix 2. Details of model development and predictor selection

Supplemental Figure 2 illustrates pipeline of the study. To build the predictive models, all variables including demographics, ICD-10 diagnostic codes, medications, and lab test results, are considered as binary digits. Ages are grouped as 10-12, 13-15, and 15-18 year groups. In addition, variables in terms of ICD-10 diagnostic codes, medications, and lab test results are set as 1 if they are present at any records before prediction window of the specific patient; otherwise 0.

For each prediction window, we randomly divided the dataset into training set (90%) and testing set (10%). Over the training set, we first performed predictor screening and variables whose corrected p-value reached significance criterion (corrected p-value < 0.1), were used as candidate predictors to develop the predictive models. We then introduced the logistic regression classifier to predict the risk of suicidal attempts and selected predictors by introducing a sequential forward selection procedure.

**Sequential forward selection.**^1,2^ Given a set $C$ of candidate predictors (after predictor screening), the sequential forward selection procedure greedily selected predictors to obtain the selected predictor set *S*. In particular,

1. We initialized the predictor set as empty, i.e., $S_{0}=\emptyset$;
2. In each step $k\in\{1, 2, 3,\ldots\}$, we selected an optimal feature $\hat{x}\in C$, such that

$\hat{x}=\arg\max J(S_{k}+x)$, $x\in C$

where, $J(S_{k}+x)$ is the prediction performance in terms of the area under the receiver operating characteristics curve (AUC) of prediction model based on predictors from $S_{k}$ plus $x$. To measure predictive performance, we introduced a 5-fold cross validation strategy and calculated the mean of the area under the receiver operating characteristics curve (AUC).

1. Update,

$S_{k}=S_{k}+\hat{x}$;

$C=C-\hat{x}$;

$k=k+1$;

1. Go back to step 2).

Of note, as the number of selected predictors increased, the AUC first raised and then declined, because too many predictors will lead to overfitting of the model, i.e., the model can well fit training set but fail to predict over test set. Therefore, we stopped the selection procedure when AUC begin to decline.

To evaluate the robustness of our predictive model over the whole population, we repeated the above selection procedures 10 times since the training-testing set split.

# Appendix 3. Calculation of contribution coefficient

For each predictor category $c$ comprised of a subset of predictors $S_{c}$, the contribution coefficient is defined by

$$\sum_{x\in S_{c}} |\beta_{x}|$$

where, $\beta_{x}$ is the coefficient of predictor $x$ by logistic regression. For each prediction window, the contribution coefficient was standardized by the maximal value.

# References

1. Ferri FJ, Pudil P, Hatef M, Kittler J. Comparative study of techniques for large-scale feature selection. InMachine Intelligence and Pattern Recognition 1994 Jan 1 (Vol. 16, pp. 403-413). North-Holland.
2. Raschka S. MLxtend: providing machine learning and data science utilities and extensions to Python's scientific computing stack. Journal of open source software. 2018 Apr 22;3(24):638.

**Table 1. Identification of suicide-related admissions using ICD-9 codes.** If either of the three inclusive criterion is identified, then a record is identified as indicating a suicide behavior. **Abbreviations**: ICD-9 indicates International Classification of Diseases, Ninth Revision.

| **Code Type** | **ICD-9 Codes** |
| --- | --- |
| Suicide Attempts | E950-E958 (intentional self-harm) |
| Suicide V-Code | V62.84 (suicide ideation) --AND SAME VISIT-- 870-899, 960-989 |
| Suicide Algorithm | 881, 960-979, 980-989, 994.7 ----AND SAME VISIT---- 293.83, 296.20-296.36, 296.82, 296.90, 298.0, 300.4, 309.0-309.1, 311, 296.00-296.06, 296.1-296.14, 296.40-296.89, 296.99, 301.13, 301, 290.8-290.9, 295, 297, 298.1-298.9, 299, 301.20-301.22, 780.1, 309.2-309.9 |

**Table 2. Longitudinal characteristics of the study population.**

**Abbreviations**: CI indicates confidence interval.

| **Characteristics** | **Positive Subjects**  **(n=180)** | **Negative subjects**  **(n=41,541)** |
| --- | --- | --- |
| Patients with records censored by prediction window, n (%) |  |  |
| Prediction window = 0 | 180 (100) | 41,541 (100) |
| Prediction window = 7 | 177 (98.3) | 19,351 (46.6) |
| Prediction window = 14 | 175 (97.2) | 18,844 (45.4) |
| Prediction window = 30 | 167 (92.8) | 17,620 (42.4) |
| Prediction window = 60 | 149 (82.8) | 16,002 (38.5) |
| Prediction window = 90 | 139 (77.2) | 14,925 (35.9) |
| Prediction window = 180 | 114 (63.3) | 12,452 (30.0) |
| Prediction window = 270 | 83 (46.1) | 10,337 (24.9) |
| Prediction window = 365 | 60 (33.3) | 8,306 (20.0) |
| Time span of longitudinal records, mean days (95% CI) | 146.8 (119.1, 174.6) | 159.5 (157.1, 161.9) |

**Table 3. Top 30 predictors of 0-day prediction window**. [I] denotes ICD diagnosis code; [D] denotes demographics; [M] denotes medication; [T] denotes lab test result. For lab test results, U=Unspecified; H=High; L=Low; A=Abnormal. Frequency and rank denote the frequency and rank that a predictor was selected by our model. **Abbreviations**: OR indicates odds ratio; CI indicates confidence interval.

| **Variable** | **Positive exposed / Positive non-exposed** | **Negative exposed / Negative non-exposed** | **OR (95% CI)** | **Selection frequency** | **Average selection rank** |
| --- | --- | --- | --- | --- | --- |
| [D]Age 10-12 | 10 / 170 | 14064 / 27477 | 0.12 (0.06, 0.22) | 1 | 2 |
| [I]R45 (Symptoms and signs involving emotional state) | 78 / 102 | 1459 / 40082 | 20.91 (15.64, 28.22) | 1 | 2 |
| [I]F32 (Major depressive disorder, single episode) | 80 / 100 | 1885 / 39656 | 16.78 (12.55, 22.65) | 1 | 2.8 |
| [D]Female Sex | 143 / 37 | 20610 / 20931 | 3.94 (2.75, 5.64) | 1 | 3.2 |
| [M]SERTRALINE (Selective serotonin reuptake inhibitors (SSRI)) | 32 / 148 | 682 / 40859 | 12.94 (8.76, 19.11) | 0.8 | 8.62 |
| [M]HYDROXYZINE PAMOATE (Treatment of itchiness, anxiety, and nausea) | 8 / 172 | 95 / 41446 | 20.29 (9.68, 42.52) | 0.8 | 10.88 |
| [T]CULTURE, URINE{U} (Routine tests) | 24 / 156 | 1812 / 39729 | 3.39 (2.18, 5.21) | 0.7 | 8 |
| [M]ETHINYL ESTRADIOL (Birth control pills) | 6 / 174 | 298 / 41243 | 4.76 (2.10, 10.80) | 0.7 | 13.86 |
| [T]ACETAMINOPHEN LEVEL{U} (Drug tests) | 14 / 166 | 281 / 41260 | 12.43 (7.10, 21.54) | 0.6 | 8.83 |
| [T]URINALYSIS WITH MICROSCOPIC{H} (Routine tests) | 23 / 157 | 1681 / 39860 | 3.49 (2.23, 5.42) | 0.6 | 10.17 |
| [T]POCT PREGNANCY, URINE{U} (Pregnancy) | 8 / 172 | 509 / 41032 | 3.74 (1.84, 7.69) | 0.6 | 12.5 |
| [I]F90 (Attention-deficit hyperactivity disorders) | 22 / 158 | 1729 / 39812 | 3.22 (2.05, 5.00) | 0.5 | 5.2 |
| [M]LORAZEPAM (Benzodiazepine and anticonvulsant medication) | 23 / 157 | 961 / 40580 | 6.17 (3.97, 9.58) | 0.5 | 7.6 |
| [T]LACTATE DEHYDROGENASE{U} (Routine tests) | 21 / 159 | 2032 / 39509 | 2.56 (1.63, 4.06) | 0.5 | 12.6 |
| [T]MDMA (ECSTASY), URINE{U} (Drug tests) | 43 / 137 | 809 / 40732 | 15.80 (11.13, 22.42) | 0.4 | 7.75 |
| [I]F91 (Conduct disorders) | 40 / 140 | 1414 / 40127 | 8.08 (5.70, 11.59) | 0.4 | 10.25 |
| [M]ESCITALOPRAM (Selective serotonin reuptake inhibitors (SSRI)) | 23 / 157 | 352 / 41189 | 17.12 (10.91, 26.84) | 0.4 | 11.5 |
| [M]CITALOPRAM (Selective serotonin reuptake inhibitors (SSRI)) | 8 / 172 | 269 / 41272 | 7.17 (3.49, 14.59) | 0.4 | 16.5 |
| [M]FLUOXETINE (Selective serotonin reuptake inhibitors (SSRI)) | 25 / 155 | 631 / 40910 | 10.49 (6.82, 16.12) | 0.3 | 10.33 |
| [T]POCT URINALYSIS DIPSTICK{A} (Routine tests) | 28 / 152 | 3036 / 38505 | 2.34 (1.55, 3.49) | 0.3 | 12 |
| [M]AZITHROMYCIN (Bacterial infections) | 9 / 171 | 519 / 41022 | 4.18 (2.12, 8.17) | 0.3 | 15.33 |
| ICD10:Z90 | 5 / 175 | 306 / 41235 | 3.86 (1.57, 9.39) | 0.3 | 16 |
| [M]HYDROXYZINE HCL (Antihistamine) | 12 / 168 | 258 / 41283 | 11.47 (6.30, 20.70) | 0.3 | 18.33 |
| [T]URINALYSIS WITH MICROSCOPIC{U} (Routine tests) | 33 / 147 | 3285 / 38256 | 2.61 (1.79, 3.82) | 0.2 | 6 |
| [D]Age 16-18 | 89 / 91 | 12405 / 29136 | 2.29 (1.72, 3.06) | 0.2 | 7.5 |
| [I]F31 (Bipolar disorder) | 10 / 170 | 231 / 41310 | 10.49 (5.47, 20.09) | 0.2 | 10 |
| [I]F41 (Other anxiety disorders) | 43 / 137 | 1905 / 39636 | 6.55 (4.62, 9.21) | 0.2 | 10.5 |
| [I]Z86 (Personal history of certain other diseases) | 21 / 159 | 1085 / 40456 | 4.90 (3.13, 7.77) | 0.2 | 11.5 |
| [M]CHOLECALCIFEROL (Vitamin) | 13 / 167 | 700 / 40841 | 4.53 (2.56, 8.00) | 0.2 | 11.5 |
| [M]VENLAFAXINE (Major depressive disorder) | 7 / 173 | 73 / 41468 | 22.87 (10.49, 50.40) | 0.2 | 13 |

**Table 4. Top 30 predictors of 7-day prediction window.** [I] denotes ICD diagnosis code; [D] denotes demographics; [M] denotes medication; [T] denotes lab test result. For lab test results, U=Unspecified; H=High; L=Low; A=Abnormal. Frequency and rank denote the frequency and rank that a predictor was selected by our model. **Abbreviations**: OR indicates odds ratio; CI indicates confidence interval.

| **Variable** | **Positive exposed / Positive non-exposed** | **Negative exposed / Negative non-exposed** | **OR (95% CI)** | **Selection frequency** | **Average selection rank** |
| --- | --- | --- | --- | --- | --- |
| [I]R45 (Symptoms and signs involving emotional state) | 78 / 99 | 929 / 18422 | 15.64 (11.47, 21.12) | 1 | 2.2 |
| [D]Age 10-12 | 8 / 169 | 6362 / 12989 | 0.10 (0.05, 0.20) | 1 | 2.3 |
| [D]Female Sex | 143 / 34 | 9832 / 9519 | 4.06 (2.80, 5.93) | 1 | 2.7 |
| [I]F32 (Major depressive disorder, single episode) | 80 / 97 | 1205 / 18146 | 12.43 (9.21, 16.78) | 1 | 2.8 |
| [M]ESCITALOPRAM (Selective serotonin reuptake inhibitors (SSRI)) | 23 / 154 | 231 / 19120 | 12.30 (7.85, 19.49) | 0.7 | 6.86 |
| [M]SERTRALINE (Selective serotonin reuptake inhibitors (SSRI)) | 31 / 146 | 451 / 18900 | 8.94 (5.99, 13.20) | 0.7 | 9 |
| [M]ETHINYL ESTRADIOL (Birth control pills) | 6 / 171 | 194 / 19157 | 3.46 (1.52, 7.92) | 0.7 | 12.29 |
| [T]URINALYSIS WITH MICROSCOPIC{U} (Routine tests) | 33 / 144 | 2197 / 17154 | 1.79 (1.22, 2.61) | 0.6 | 5.17 |
| [M]HYDROXYZINE PAMOATE (Treatment of itchiness, anxiety, and nausea) | 7 / 170 | 73 / 19278 | 10.91 (4.95, 24.05) | 0.6 | 11.33 |
| [M]GUANFACINE (Treatment of ADHD and high blood pressure) | 14 / 163 | 422 / 18929 | 3.86 (2.20, 6.69) | 0.5 | 7.8 |
| [T]ACETAMINOPHEN LEVEL{U} (Drug tests) | 14 / 163 | 180 / 19171 | 9.12 (5.21, 16.12) | 0.5 | 9.6 |
| [M]FLUOXETINE (Selective serotonin reuptake inhibitors (SSRI)) | 25 / 152 | 466 / 18885 | 6.69 (4.31, 10.28) | 0.5 | 11.4 |
| [T]CULTURE, URINE{U} (Routine tests) | 24 / 153 | 1296 / 18055 | 2.18 (1.42, 3.39) | 0.4 | 5.5 |
| [I]F39 (Unspecified mood [affective] disorder) | 34 / 143 | 508 / 18843 | 8.85 (5.99, 12.94) | 0.4 | 6.5 |
| [M]TRAZODONE (Serotonin modulator) | 19 / 158 | 218 / 19133 | 10.59 (6.42, 17.29) | 0.4 | 7.25 |
| [T]POCT, PREGNANCY, URINE(ED&IP){U} (Pregnancy) | 84 / 93 | 3399 / 15952 | 4.22 (3.16, 5.70) | 0.4 | 10.75 |
| [T]SALICYLATE LEVEL{L} (Drug tests) | 14 / 163 | 192 / 19159 | 8.58 (4.85, 15.03) | 0.4 | 17 |
| [I]M25 (Other joint disorder, not elsewhere classified) | 9 / 168 | 3127 / 16224 | 0.28 (0.14, 0.54) | 0.3 | 9.33 |
| [I]F31 (Bipolar disorder) | 10 / 167 | 184 / 19167 | 6.23 (3.25, 12.06) | 0.3 | 10 |
| [T]OPIATE, URINE, QUALITATIVE{U} (Drug test) | 54 / 123 | 931 / 18420 | 8.67 (6.30, 12.06) | 0.3 | 10.33 |
| [M]CLONAZEPAM (Seizures, panic disorder, and the movement disorder) | 8 / 169 | 210 / 19141 | 4.31 (2.10, 8.85) | 0.3 | 15.33 |
| [I]Z72 (Problems related to lifestyle) | 10 / 167 | 157 / 19194 | 7.32 (3.78, 14.15) | 0.3 | 15.67 |
| [T]POCT PREGNANCY, URINE{U} (Pregnancy) | 8 / 169 | 342 / 19009 | 2.64 (1.28, 5.37) | 0.2 | 8 |
| [M]LORAZEPAM (Benzodiazepine and anticonvulsant medication) | 22 / 155 | 764 / 18587 | 3.46 (2.20, 5.42) | 0.2 | 10 |
| [T]CULTURE, URINE{A} (Routine tests) | 11 / 166 | 433 / 18918 | 2.89 (1.57, 5.37) | 0.2 | 11.5 |
| [M]PROZAC (Antidepressant) | 12 / 165 | 72 / 19279 | 19.49 (10.38, 36.60) | 0.2 | 12 |
| [M]RISPERIDONE (Atypical antipsychotics) | 13 / 164 | 273 / 19078 | 5.53 (3.10, 9.87) | 0.2 | 12.5 |
| [M]AZITHROMYCIN (Bacterial infections) | 9 / 168 | 384 / 18967 | 2.64 (1.34, 5.21) | 0.2 | 13 |
| [T]AMPHETAMINE, URINE, QUALITATIVE{U} (Drug test) | 56 / 121 | 902 / 18449 | 9.49 (6.82, 13.07) | 0.2 | 13.5 |
| [I]F93 (Emotional disorders with onset specific to childhood) | 5 / 172 | 104 / 19247 | 5.37 (2.16, 13.33) | 0.2 | 14 |

**Table 5. Top 30 predictors of 14-day prediction window.** [I] denotes ICD diagnosis code; [D] denotes demographics; [M] denotes medication; [T] denotes lab test result. For lab test results, U=Unspecified; H=High; L=Low; A=Abnormal. Frequency and rank denote the frequency and rank that a predictor was selected by our model. **Abbreviations**: OR indicates odds ratio; CI indicates confidence interval.

| **Variable** | **Positive exposed / Positive non-exposed** | **Negative exposed / Negative non-exposed** | **OR (95% CI)** | **Selection frequency** | **Average selection rank** |
| --- | --- | --- | --- | --- | --- |
| [I]R45 (Symptoms and signs involving emotional state) | 76 / 99 | 919 / 17925 | 15.03 (11.02, 20.29) | 1 | 1.3 |
| [D]Age 10-12 | 8 / 167 | 6186 / 12658 | 0.10 (0.05, 0.20) | 1 | 2.2 |
| [D]Female Sex | 141 / 34 | 9610 / 9234 | 3.97 (2.75, 5.81) | 1 | 2.8 |
| [I]F32 (Major depressive disorder, single episode) | 78 / 97 | 1185 / 17659 | 11.94 (8.85, 16.28) | 1 | 3.7 |
| [T]URINALYSIS WITH MICROSCOPIC{U} (Routine tests) | 33 / 142 | 2156 / 16688 | 1.80 (1.23, 2.64) | 0.8 | 5.25 |
| [M]ESCITALOPRAM (Selective serotonin reuptake inhibitors (SSRI)) | 23 / 152 | 227 / 18617 | 12.43 (7.85, 19.69) | 0.8 | 7.25 |
| [M]SERTRALINE (Selective serotonin reuptake inhibitors (SSRI)) | 30 / 145 | 442 / 18402 | 8.58 (5.75, 12.94) | 0.6 | 10 |
| [T]ACETAMINOPHEN LEVEL{U} (Drug tests) | 14 / 161 | 180 / 18664 | 9.03 (5.10, 15.80) | 0.6 | 10.17 |
| [I]M25 (Other joint disorder, not elsewhere classified) | 9 / 166 | 3047 / 15797 | 0.28 (0.14, 0.55) | 0.5 | 7.8 |
| [M]HYDROXYZINE PAMOATE (Treatment of itchiness, anxiety, and nausea) | 7 / 168 | 72 / 18772 | 10.91 (4.90, 24.05) | 0.5 | 9.4 |
| [M]GUANFACINE (Treatment of ADHD and high blood pressure) | 14 / 161 | 419 / 18425 | 3.82 (2.20, 6.69) | 0.5 | 10 |
| [T]SALICYLATE LEVEL{L} (Drug tests) | 14 / 161 | 192 / 18652 | 8.41 (4.81, 14.88) | 0.5 | 14.6 |
| [M]LORAZEPAM (Benzodiazepine and anticonvulsant medication) | 22 / 153 | 750 / 18094 | 3.46 (2.20, 5.47) | 0.5 | 14.8 |
| [M]TRAZODONE (Serotonin modulator) | 19 / 156 | 214 / 18630 | 10.59 (6.49, 17.46) | 0.4 | 9.75 |
| [M]PROZAC (Antidepressant) | 12 / 163 | 70 / 18774 | 19.69 (10.49, 36.97) | 0.4 | 11 |
| [I]F91 (Conduct disorders) | 38 / 137 | 1030 / 17814 | 4.81 (3.32, 6.89) | 0.4 | 11.5 |
| [M]RISPERIDONE (Atypical antipsychotics) | 13 / 162 | 270 / 18574 | 5.53 (3.10, 9.87) | 0.4 | 11.75 |
| [M]ZOLOFT (Antidepressant) | 9 / 166 | 96 / 18748 | 10.59 (5.26, 21.33) | 0.4 | 13.75 |
| [M]ARIPIPRAZOLE (Antipsychotic medication) | 24 / 151 | 435 / 18409 | 6.75 (4.31, 10.49) | 0.3 | 10.33 |
| [I]F31 (Bipolar disorder) | 10 / 165 | 180 / 18664 | 6.30 (3.25, 12.06) | 0.3 | 10.67 |
| [I]R44 (Other symptoms and signs involving general sensations and perceptions) | 7 / 168 | 101 / 18743 | 7.77 (3.53, 16.95) | 0.3 | 11.33 |
| [T]GC/CHLAMYDIA{U} (Routine tests) | 14 / 161 | 365 / 18479 | 4.39 (2.53, 7.69) | 0.3 | 13.33 |
| [M]ETHINYL ESTRADIOL (Birth control pills) | 6 / 169 | 190 / 18654 | 3.49 (1.52, 8.00) | 0.3 | 15 |
| [M]HYDROXYZINE HCL (Antihistamine) | 12 / 163 | 189 / 18655 | 7.24 (3.97, 13.33) | 0.3 | 15 |
| [D]F63 (Impulse disorders) | 6 / 169 | 158 / 18686 | 4.18 (1.84, 9.58) | 0.3 | 15.67 |
| [I]Z72 (Problems related to lifestyle) | 10 / 165 | 156 / 18688 | 7.24 (3.74, 14.01) | 0.3 | 17 |
| [D]Age 16-18 | 88 / 87 | 5938 / 12906 | 2.20 (1.63, 2.97) | 0.2 | 7 |
| [T]CULTURE, URINE{U} (Routine tests) | 24 / 151 | 1271 / 17573 | 2.20 (1.42, 3.39) | 0.2 | 7 |
| [I]F48 (Other nonpsychotic mental disorders) | 12 / 163 | 122 / 18722 | 11.25 (6.11, 20.91) | 0.2 | 9.5 |
| [T]POCT, PREGNANCY, URINE(ED&IP){U} (Pregnancy) | 84 / 91 | 3321 / 15523 | 4.31 (3.19, 5.81) | 0.2 | 10.5 |

**Table 6. Top 30 predictors of 30-day prediction window.** [I] denotes ICD diagnosis code; [D] denotes demographics; [M] denotes medication; [T] denotes lab test result. For lab test results, U=Unspecified; H=High; L=Low; A=Abnormal. Frequency and rank denote the frequency and rank that a predictor was selected by our model. **Abbreviations**: OR indicates odds ratio; CI indicates confidence interval.

| **Variable** | **Positive exposed / Positive non-exposed** | **Negative exposed / Negative non-exposed** | **OR (95% CI)** | **Selection frequency** | **Average selection rank** |
| --- | --- | --- | --- | --- | --- |
| [I]F32 (Major depressive disorder, single episode) | 76 / 91 | 1150 / 16470 | 11.94 (8.76, 16.28) | 1 | 1.9 |
| [D]Age 10-12 | 8 / 159 | 5771 / 11849 | 0.10 (0.05, 0.21) | 1 | 2.3 |
| [D]Female Sex | 135 / 32 | 8977 / 8643 | 4.06 (2.77, 5.99) | 1 | 2.7 |
| [I]R45 (Symptoms and signs involving emotional state) | 71 / 96 | 897 / 16723 | 13.74 (10.07, 18.92) | 1 | 3.1 |
| [M]ESCITALOPRAM (Selective serotonin reuptake inhibitors (SSRI)) | 21 / 146 | 220 / 17400 | 11.36 (7.10, 18.36) | 0.9 | 7 |
| [T]ACETAMINOPHEN LEVEL{U} (Drug tests) | 14 / 153 | 174 / 17446 | 9.21 (5.21, 16.12) | 0.8 | 8.62 |
| [M]SERTRALINE (Selective serotonin reuptake inhibitors (SSRI)) | 30 / 137 | 426 / 17194 | 8.85 (5.87, 13.33) | 0.7 | 9.57 |
| [I]M25 (Other joint disorder, not elsewhere classified) | 9 / 158 | 2811 / 14809 | 0.30 (0.15, 0.59) | 0.6 | 10.33 |
| [T]URINALYSIS WITH MICROSCOPIC{U} (Routine tests) | 33 / 134 | 2079 / 15541 | 1.84 (1.26, 2.69) | 0.5 | 8.2 |
| [M]RISPERIDONE (Atypical antipsychotics) | 13 / 154 | 263 / 17357 | 5.58 (3.13, 9.97) | 0.4 | 8.75 |
| [M]GUANFACINE (Treatment of ADHD and high blood pressure) | 14 / 153 | 409 / 17211 | 3.86 (2.20, 6.69) | 0.4 | 10 |
| [M]ETHINYL ESTRADIOL (Birth control pills) | 6 / 161 | 189 / 17431 | 3.42 (1.51, 7.85) | 0.4 | 11.5 |
| [M]HYDROXYZINE PAMOATE (Treatment of itchiness, anxiety, and nausea) | 7 / 160 | 67 / 17553 | 11.47 (5.21, 25.28) | 0.4 | 12.5 |
| [T]POCT PREGNANCY, URINE{U} (Pregnancy) | 8 / 159 | 315 / 17305 | 2.77 (1.35, 5.70) | 0.4 | 12.5 |
| [T]SALICYLATE LEVEL{L} (Drug tests) | 14 / 153 | 186 / 17434 | 8.58 (4.85, 15.18) | 0.4 | 14 |
| [M]VENLAFAXINE (Major depressive disorder) | 7 / 160 | 51 / 17569 | 15.03 (6.75, 33.78) | 0.4 | 14.75 |
| [D]Age 16-18 | 84 / 83 | 5610 / 12010 | 2.16 (1.60, 2.94) | 0.3 | 5 |
| [I]F39 (Unspecified mood [affective] disorder) | 33 / 134 | 490 / 17130 | 8.58 (5.81, 12.68) | 0.3 | 7.33 |
| [T]POCT, PREGNANCY, URINE(ED&IP){U} (Pregnancy) | 81 / 86 | 3169 / 14451 | 4.31 (3.16, 5.81) | 0.3 | 9 |
| [M]PROZAC (Antidepressant) | 12 / 155 | 68 / 17552 | 19.89 (10.59, 37.71) | 0.3 | 11 |
| [M]TRAZODONE (Serotonin modulator) | 18 / 149 | 209 / 17411 | 10.07 (6.05, 16.78) | 0.3 | 11 |
| [M]QUETIAPINE (Atypical antipsychotic) | 15 / 152 | 244 / 17376 | 7.03 (4.06, 12.18) | 0.3 | 13.67 |
| [T]GC/CHLAMYDIA{U} (Routine tests) | 14 / 153 | 356 / 17264 | 4.44 (2.53, 7.77) | 0.2 | 7 |
| [T]CULTURE, URINE{U} (Routine tests) | 23 / 144 | 1233 / 16387 | 2.12 (1.36, 3.32) | 0.2 | 7.5 |
| [T]URINALYSIS WITH MICROSCOPIC{H} (Routine tests) | 23 / 144 | 1106 / 16514 | 2.39 (1.52, 3.71) | 0.2 | 10.5 |
| [I]F90 (Attention-deficit hyperactivity disorders) | 20 / 147 | 1202 / 16418 | 1.86 (1.16, 2.97) | 0.2 | 11.5 |
| [T]BENZODIAZEPINE SCREEN, URINE{U} (Drug tests) | 57 / 110 | 875 / 16745 | 9.87 (7.17, 13.74) | 0.2 | 11.5 |
| [M]MELATONIN (Hormone that regulates sleep-wake cycles) | 13 / 154 | 528 / 17092 | 2.75 (1.54, 4.85) | 0.2 | 12.5 |
| [M]FLUOXETINE (Selective serotonin reuptake inhibitors (SSRI)) | 25 / 142 | 444 / 17176 | 6.82 (4.39, 10.49) | 0.2 | 13 |
| [T]OXYCODONE, URINE{U} (Drug tests) | 15 / 152 | 131 / 17489 | 13.20 (7.54, 23.10) | 0.2 | 15.5 |

**Table 7. Top 30 predictors of 60-day prediction window.** [I] denotes ICD diagnosis code; [D] denotes demographics; [M] denotes medication; [T] denotes lab test result. For lab test results, U=Unspecified; H=High; L=Low; A=Abnormal. **Abbreviations**: OR indicates odds ratio; CI indicates confidence interval.

| **Variable** | **Positive exposed / Positive non-exposed** | **Negative exposed / Negative non-exposed** | **OR (95% CI)** | **Selection frequency** | **Average selection rank** |
| --- | --- | --- | --- | --- | --- |
| [I]F32 (Major depressive disorder, single episode) | 70 / 79 | 1102 / 14900 | 11.94 (8.67, 16.61) | 1 | 2.2 |
| [D]Age 10-12 | 7 / 142 | 5245 / 10757 | 0.10 (0.05, 0.22) | 1 | 2.5 |
| [D]Female Sex | 120 / 29 | 8180 / 7822 | 3.97 (2.64, 5.93) | 1 | 2.5 |
| [I]R45 (Symptoms and signs involving emotional state) | 67 / 82 | 856 / 15146 | 14.44 (10.38, 20.09) | 1 | 2.8 |
| [M]ESCITALOPRAM (Selective serotonin reuptake inhibitors (SSRI)) | 19 / 130 | 211 / 15791 | 10.91 (6.62, 17.99) | 1 | 7.4 |
| [M]SERTRALINE (Selective serotonin reuptake inhibitors (SSRI)) | 29 / 120 | 400 / 15602 | 9.39 (6.23, 14.30) | 1 | 7.8 |
| [M]ETHINYL ESTRADIOL (Birth control pills) | 6 / 143 | 177 / 15825 | 3.74 (1.63, 8.58) | 0.9 | 12.11 |
| [T]ACETAMINOPHEN LEVEL{U} (Drug tests) | 13 / 136 | 161 / 15841 | 9.39 (5.21, 16.95) | 0.6 | 7.83 |
| [I]M25 (Other joint disorder, not elsewhere classified) | 9 / 140 | 2518 / 13484 | 0.34 (0.18, 0.68) | 0.6 | 10.5 |
| [T]CULTURE, URINE{U} (Routine tests) | 23 / 126 | 1189 / 14813 | 2.27 (1.45, 3.56) | 0.5 | 7.4 |
| [D]Age 16-18 | 74 / 75 | 5146 / 10856 | 2.08 (1.51, 2.89) | 0.4 | 6 |
| [T]URINALYSIS WITH MICROSCOPIC{U} (Routine tests) | 32 / 117 | 1991 / 14011 | 1.92 (1.30, 2.86) | 0.4 | 6 |
| [M]TRAZODONE (Serotonin modulator) | 18 / 131 | 202 / 15800 | 10.70 (6.42, 17.99) | 0.4 | 8.25 |
| [I]F31 (Bipolar disorder) | 10 / 139 | 169 / 15833 | 6.75 (3.49, 13.07) | 0.3 | 9.33 |
| [I]Z72 (Problems related to lifestyle) | 8 / 141 | 149 / 15853 | 6.05 (2.92, 12.55) | 0.3 | 14 |
| [M]ARIPIPRAZOLE (Antipsychotic medication) | 24 / 125 | 412 / 15590 | 7.24 (4.66, 11.36) | 0.3 | 14 |
| [M]GUANFACINE (Treatment of ADHD and high blood pressure) | 13 / 136 | 396 / 15606 | 3.78 (2.12, 6.69) | 0.3 | 14.33 |
| [M]HYDROXYZINE HCL (Antihistamine) | 11 / 138 | 177 / 15825 | 7.10 (3.78, 13.46) | 0.3 | 16 |
| [T]MDMA (ECSTASY), URINE{U} (Drug tests) | 39 / 110 | 519 / 15483 | 10.59 (7.24, 15.33) | 0.2 | 9 |
| [M]QUETIAPINE (Atypical antipsychotic) | 15 / 134 | 238 / 15764 | 7.39 (4.26, 12.81) | 0.2 | 9.5 |
| [I]F39 (Unspecified mood [affective] disorder) | 29 / 120 | 473 / 15529 | 7.92 (5.26, 12.06) | 0.2 | 10.5 |
| [M]ZOLOFT (Antidepressant) | 7 / 142 | 88 / 15914 | 8.94 (4.06, 19.49) | 0.2 | 10.5 |
| [M]FLUOXETINE (Selective serotonin reuptake inhibitors (SSRI)) | 22 / 127 | 427 / 15575 | 6.30 (3.97, 10.07) | 0.2 | 11 |
| [T]BENZODIAZEPINE SCREEN, URINE{U} (Drug tests) | 54 / 95 | 832 / 15170 | 10.38 (7.39, 14.59) | 0.2 | 12 |
| [T]URINALYSIS WITH MICROSCOPIC{H} (Routine tests) | 22 / 127 | 1064 / 14938 | 2.44 (1.54, 3.86) | 0.2 | 12 |
| [M]RISPERIDONE (Atypical antipsychotics) | 12 / 137 | 243 / 15759 | 5.70 (3.10, 10.38) | 0.2 | 12.5 |
| [M]PROZAC (Antidepressant) | 10 / 139 | 66 / 15936 | 17.29 (8.76, 34.47) | 0.2 | 13.5 |
| [M]CITALOPRAM (Selective serotonin reuptake inhibitors (SSRI)) | 8 / 141 | 185 / 15817 | 4.85 (2.34, 10.07) | 0.2 | 13.5 |
| [T]POCT, PREGNANCY, URINE(ED&IP){U} (Pregnancy) | 77 / 72 | 2977 / 13025 | 4.66 (3.39, 6.49) | 0.2 | 15.5 |
| [T]OXYCODONE, URINE{U} (Drug tests) | 14 / 135 | 125 / 15877 | 13.20 (7.39, 23.57) | 0.2 | 16 |

**Table 8. Top 30 predictors of 90-day prediction window.** [I] denotes ICD diagnosis code; [D] denotes demographics; [M] denotes medication; [T] denotes lab test result. For lab test results, U=Unspecified; H=High; L=Low; A=Abnormal. Frequency and rank denote the frequency and rank that a predictor was selected by our model. **Abbreviations**: OR indicates odds ratio; CI indicates confidence interval.

| **Variable** | **Positive exposed / Positive non-exposed** | **Negative exposed / Negative non-exposed** | **OR (95% CI)** | **Selection frequency** | **Average selection rank** |
| --- | --- | --- | --- | --- | --- |
| [I]F32 (Major depressive disorder, single episode) | 67 / 72 | 1042 / 13883 | 12.43 (8.85, 17.46) | 1 | 2.1 |
| [D]Age 10-12 | 7 / 132 | 4914 / 10011 | 0.11 (0.05, 0.23) | 1 | 2.7 |
| [D]Female Sex | 110 / 29 | 7632 / 7293 | 3.63 (2.41, 5.47) | 1 | 2.9 |
| [I]R45 (Symptoms and signs involving emotional state) | 65 / 74 | 810 / 14115 | 15.33 (10.91, 21.54) | 0.9 | 2.67 |
| [M]ETHINYL ESTRADIOL (Birth control pills) | 6 / 133 | 164 / 14761 | 4.06 (1.77, 9.30) | 0.9 | 11.67 |
| [M]SERTRALINE (Selective serotonin reuptake inhibitors (SSRI)) | 27 / 112 | 381 / 14544 | 9.21 (5.99, 14.15) | 0.8 | 9.25 |
| [M]ESCITALOPRAM (Selective serotonin reuptake inhibitors (SSRI)) | 18 / 121 | 200 / 14725 | 10.91 (6.55, 18.36) | 0.6 | 7.5 |
| [T]ACETAMINOPHEN LEVEL{U} (Drug tests) | 11 / 128 | 152 / 14773 | 8.33 (4.44, 15.80) | 0.6 | 8.17 |
| [T]CULTURE, URINE{U} (Routine tests) | 22 / 117 | 1143 / 13782 | 2.27 (1.43, 3.60) | 0.5 | 5.4 |
| [M]TRAZODONE (Serotonin modulator) | 17 / 122 | 195 / 14730 | 10.49 (6.23, 17.81) | 0.4 | 5.75 |
| [D]Age 16-18 | 69 / 70 | 4797 / 10128 | 2.08 (1.49, 2.92) | 0.4 | 6.5 |
| [T]URINALYSIS WITH MICROSCOPIC{U} (Routine tests) | 31 / 108 | 1907 / 13018 | 1.95 (1.31, 2.92) | 0.4 | 7 |
| [I]F39 (Unspecified mood [affective] disorder) | 28 / 111 | 457 / 14468 | 8.00 (5.21, 12.18) | 0.4 | 7.75 |
| [I]F31 (Bipolar disorder) | 10 / 129 | 161 / 14764 | 7.10 (3.67, 13.74) | 0.4 | 9.75 |
| [M]FLUOXETINE (Selective serotonin reuptake inhibitors (SSRI)) | 21 / 118 | 405 / 14520 | 6.36 (3.97, 10.28) | 0.4 | 11.75 |
| [T]OXYCODONE, URINE{U} (Drug tests) | 12 / 127 | 119 / 14806 | 11.70 (6.36, 21.76) | 0.4 | 12.5 |
| [I]F91 (Conduct disorders) | 34 / 105 | 929 / 13996 | 4.85 (3.29, 7.24) | 0.3 | 10.67 |
| [M]CITALOPRAM (Selective serotonin reuptake inhibitors (SSRI)) | 8 / 131 | 182 / 14743 | 4.95 (2.39, 10.28) | 0.3 | 10.67 |
| [T]CULTURE, URINE{A} (Routine tests) | 10 / 129 | 391 / 14534 | 2.89 (1.51, 5.53) | 0.3 | 13 |
| [M]CHOLECALCIFEROL (Vitamin) | 13 / 126 | 549 / 14376 | 2.69 (1.52, 4.81) | 0.2 | 9 |
| [M]GUANFACINE (Treatment of ADHD and high blood pressure) | 12 / 127 | 383 / 14542 | 3.60 (1.97, 6.55) | 0.2 | 11 |
| [I]Z91 (Personal history of risk-factors, not elsewhere classified) | 31 / 108 | 1635 / 13290 | 2.34 (1.55, 3.49) | 0.2 | 12 |
| [M]LORAZEPAM (Benzodiazepine and anticonvulsant medication) | 19 / 120 | 678 / 14247 | 3.32 (2.03, 5.42) | 0.2 | 12 |
| [M]NORETHINDRONE (Birth control pills) | 5 / 134 | 91 / 14834 | 6.11 (2.44, 15.18) | 0.2 | 12.5 |
| [T]URINALYSIS WITH MICROSCOPIC{A} (Routine tests) | 25 / 114 | 1415 / 13510 | 2.10 (1.35, 3.25) | 0.2 | 12.5 |
| [T]SALICYLATE LEVEL{L} (Drug tests) | 11 / 128 | 161 / 14764 | 7.85 (4.18, 14.88) | 0.2 | 12.5 |
| [T]POCT, PREGNANCY, URINE(ED&IP){U} (Pregnancy) | 72 / 67 | 2809 / 12116 | 4.62 (3.32, 6.49) | 0.2 | 13 |
| [M]ARIPIPRAZOLE (Antipsychotic medication) | 23 / 116 | 391 / 14534 | 7.39 (4.66, 11.70) | 0.2 | 13.5 |
| [M]HYDROXYZINE HCL (Antihistamine) | 11 / 128 | 163 / 14762 | 7.77 (4.14, 14.73) | 0.2 | 14 |
| [T]TRICYCLIC SCREEN, URINE{U} (Drug tests) | 48 / 91 | 725 / 14200 | 10.38 (7.24, 14.73) | 0.2 | 14 |

**Table 9. Top 30 predictors of 180-day prediction window.** [I] denotes ICD diagnosis code; [D] denotes demographics; [M] denotes medication; [T] denotes lab test result. For lab test results, U=Unspecified; H=High; L=Low; A=Abnormal. Frequency and rank denote the frequency and rank that a predictor was selected by our model. **Abbreviations**: OR indicates odds ratio; CI indicates confidence interval.

| **Variable** | **Positive exposed / Positive non-exposed** | **Negative exposed / Negative non-exposed** | **OR (95% CI)** | **Selection frequency** | **Average selection rank** |
| --- | --- | --- | --- | --- | --- |
| [I]F32 (Major depressive disorder, single episode) | 57 / 57 | 902 / 11550 | 12.81 (8.85, 18.54) | 1 | 1.7 |
| [D]Age 10-12 | 5 / 109 | 4145 / 8307 | 0.09 (0.04, 0.23) | 1 | 2.8 |
| [D]Female Sex | 93 / 21 | 6363 / 6089 | 4.22 (2.64, 6.82) | 1 | 3.4 |
| [I]R45 (Symptoms and signs involving emotional state) | 54 / 60 | 700 / 11752 | 15.18 (10.38, 21.98) | 1 | 4.1 |
| [M]SERTRALINE (Selective serotonin reuptake inhibitors (SSRI)) | 23 / 91 | 328 / 12124 | 9.30 (5.81, 14.88) | 0.8 | 7.25 |
| [M]QUETIAPINE (Atypical antipsychotic) | 13 / 101 | 195 / 12257 | 8.08 (4.48, 14.73) | 0.8 | 10 |
| [T]URINALYSIS WITH MICROSCOPIC{H} (Routine tests) | 20 / 94 | 916 / 11536 | 2.69 (1.65, 4.35) | 0.7 | 5.86 |
| [T]ACETAMINOPHEN LEVEL{U} (Drug tests) | 10 / 104 | 133 / 12319 | 8.94 (4.57, 17.46) | 0.7 | 8.29 |
| [M]PROZAC (Antidepressant) | 9 / 105 | 56 / 12396 | 18.92 (9.12, 39.25) | 0.6 | 10.83 |
| [T]POCT, PREGNANCY, URINE(ED&IP){U} (Pregnancy) | 63 / 51 | 2400 / 10052 | 5.16 (3.56, 7.54) | 0.4 | 2 |
| [T]POCT URINALYSIS DIPSTICK{A} (Routine tests) | 24 / 90 | 1505 / 10947 | 1.93 (1.23, 3.06) | 0.4 | 7.75 |
| [M]RISPERIDONE (Atypical antipsychotics) | 10 / 104 | 204 / 12248 | 5.75 (2.97, 11.25) | 0.4 | 10.25 |
| [I]Z72 (Problems related to lifestyle) | 7 / 107 | 135 / 12317 | 5.99 (2.72, 13.07) | 0.4 | 11 |
| [I]F41 (Other anxiety disorders) | 29 / 85 | 994 / 11458 | 3.94 (2.56, 6.05) | 0.4 | 12 |
| [M]ARIPIPRAZOLE (Antipsychotic medication) | 20 / 94 | 329 / 12123 | 7.85 (4.76, 12.81) | 0.3 | 7.33 |
| [D]F63 (Impulse disorders) | 6 / 108 | 128 / 12324 | 5.37 (2.32, 12.43) | 0.3 | 9 |
| [I]F39 (Unspecified mood [affective] disorder) | 23 / 91 | 392 / 12060 | 7.77 (4.85, 12.43) | 0.3 | 10 |
| [M]CHOLECALCIFEROL (Vitamin) | 11 / 103 | 496 / 11956 | 2.59 (1.38, 4.81) | 0.3 | 10.67 |
| [M]ETHINYL ESTRADIOL (Birth control pills) | 5 / 109 | 139 / 12313 | 4.06 (1.63, 10.07) | 0.3 | 13 |
| [M]CITALOPRAM (Selective serotonin reuptake inhibitors (SSRI)) | 7 / 107 | 157 / 12295 | 5.10 (2.34, 11.13) | 0.3 | 13.67 |
| [T]SALICYLATE LEVEL{L} (Drug tests) | 10 / 104 | 140 / 12312 | 8.41 (4.31, 16.44) | 0.3 | 14 |
| [I]F34 (Persistent mood [affective] disorders) | 13 / 101 | 290 / 12162 | 5.42 (3.00, 9.78) | 0.2 | 8.5 |
| [T]GC/CHLAMYDIA{U} (Routine tests) | 11 / 103 | 282 / 12170 | 4.62 (2.46, 8.67) | 0.2 | 9.5 |
| [M]GUANFACINE (Treatment of ADHD and high blood pressure) | 11 / 103 | 338 / 12114 | 3.82 (2.03, 7.17) | 0.2 | 11.5 |
| [T]OXYCODONE SCREEN, REFLEX SEMI-QUANTITATIVE{U} (Drug tests) | 19 / 95 | 218 / 12234 | 11.25 (6.75, 18.73) | 0.2 | 12 |
| [T]BARBITURATE SCREEN, URINE{U} (Drug tests) | 36 / 78 | 563 / 11889 | 9.78 (6.49, 14.59) | 0.2 | 13.5 |
| [D]Age 16-18 | 54 / 60 | 4017 / 8435 | 1.90 (1.31, 2.75) | 0.2 | 14 |
| [T]TRICYCLIC SCREEN, URINE{U} (Drug tests) | 40 / 74 | 612 / 11840 | 10.49 (7.03, 15.49) | 0.2 | 15.5 |
| [I]F33 (Major depressive disorder, recurrent) | 22 / 92 | 214 / 12238 | 13.74 (8.41, 22.20) | 0.2 | 16 |
| [T]URINALYSIS WITH MICROSCOPIC{U} (Routine tests) | 27 / 87 | 1699 / 10753 | 1.97 (1.27, 3.03) | 0.1 | 5 |

**Table 10. Top 20 predictors of 270-day prediction window.** [I] denotes ICD diagnosis code; [D] denotes demographics; [M] denotes medication; [T] denotes lab test result. For lab test results, U=Unspecified; H=High; L=Low; A=Abnormal. Frequency and rank denote the frequency and rank that a predictor was selected by our model. **Abbreviations**: OR indicates odds ratio; CI indicates confidence interval.

| **Variable** | **Positive exposed / Positive non-exposed** | **Negative exposed / Negative non-exposed** | **OR (95% CI)** | **Selection frequency** | **Average selection rank** |
| --- | --- | --- | --- | --- | --- |
| [I]R45 (Symptoms and signs involving emotional state) | 40 / 43 | 594 / 9743 | 15.33 (9.87, 23.57) | 1 | 1 |
| [D]Age 10-12 | 2 / 81 | 3461 / 6876 | 0.05 (0.01, 0.20) | 1 | 2.8 |
| [D]Female Sex | 68 / 15 | 5253 / 5084 | 4.39 (2.51, 7.69) | 1 | 2.8 |
| [I]F32 (Major depressive disorder, single episode) | 39 / 44 | 760 / 9577 | 11.13 (7.24, 17.29) | 0.9 | 5.89 |
| [M]CHOLECALCIFEROL (Vitamin) | 11 / 72 | 444 / 9893 | 3.39 (1.79, 6.49) | 0.6 | 7.67 |
| [M]ARIPIPRAZOLE (Antipsychotic medication) | 14 / 69 | 287 / 10050 | 7.10 (3.94, 12.81) | 0.6 | 8.67 |
| [T]CULTURE, URINE{U} (Routine tests) | 15 / 68 | 890 / 9447 | 2.34 (1.34, 4.10) | 0.5 | 5.2 |
| [M]SERTRALINE (Selective serotonin reuptake inhibitors (SSRI)) | 16 / 67 | 284 / 10053 | 8.41 (4.85, 14.73) | 0.5 | 9 |
| [T]GC/CHLAMYDIA{U} (Routine tests) | 9 / 74 | 250 / 10087 | 4.90 (2.44, 9.87) | 0.4 | 5.5 |
| [I]F31 (Bipolar disorder) | 8 / 75 | 128 / 10209 | 8.50 (4.01, 17.99) | 0.4 | 7.25 |
| [T]OXYCODONE, URINE{U} (Drug tests) | 8 / 75 | 91 / 10246 | 12.06 (5.64, 25.53) | 0.4 | 10.25 |
| [D]Age 16-18 | 40 / 43 | 3332 / 7005 | 1.95 (1.27, 3.00) | 0.3 | 2 |
| [M]ESCITALOPRAM (Selective serotonin reuptake inhibitors (SSRI)) | 12 / 71 | 141 / 10196 | 12.18 (6.49, 23.10) | 0.3 | 6.67 |
| [M]GUANFACINE (Treatment of ADHD and high blood pressure) | 8 / 75 | 297 / 10040 | 3.60 (1.72, 7.54) | 0.3 | 8.67 |
| [T]ACETAMINOPHEN LEVEL{U} (Drug tests) | 6 / 77 | 109 / 10228 | 7.32 (3.13, 17.12) | 0.3 | 9 |
| [M]MELATONIN (Hormone that regulates sleep-wake cycles) | 9 / 74 | 364 / 9973 | 3.32 (1.65, 6.69) | 0.3 | 9.67 |
| [I]F41 (Other anxiety disorders) | 21 / 62 | 851 / 9486 | 3.78 (2.29, 6.23) | 0.3 | 12 |
| [M]ZOLOFT (Antidepressant) | 5 / 78 | 50 / 10287 | 13.20 (5.10, 34.12) | 0.2 | 5 |
| [T]OPIATE, URINE, QUALITATIVE{U} (Drug test) | 30 / 53 | 574 / 9763 | 9.58 (6.11, 15.18) | 0.2 | 5.5 |
| [I]F48 (Other nonpsychotic mental disorders) | 8 / 75 | 100 / 10237 | 10.91 (5.16, 23.34) | 0.2 | 9 |

**Table 11. Top 20 predictors of 365-day prediction window.** [I] denotes ICD diagnosis code; [D] denotes demographics; [M] denotes medication; [T] denotes lab test result. For lab test results, U=Unspecified; H=High; L=Low; A=Abnormal. Frequency and rank denote the frequency and rank that a predictor was selected by our model. **Abbreviations**: OR indicates odds ratio; CI indicates confidence interval.

| **Variable** | **Positive exposed / Positive non-exposed** | **Negative exposed / Negative non-exposed** | **OR (95% CI)** | **Selection frequency** | **Average selection rank** |
| --- | --- | --- | --- | --- | --- |
| [D]Age 10-12 | 1 / 59 | 2812 / 5494 | 0.03 (0.00, 0.24) | 1 | 3.5 |
| [D]Female Sex | 49 / 11 | 4235 / 4071 | 4.26 (2.23, 8.25) | 1 | 4 |
| [I]R45 (Symptoms and signs involving emotional state) | 29 / 31 | 482 / 7824 | 15.18 (9.12, 25.28) | 0.9 | 1 |
| [T]CULTURE, URINE{U} (Routine tests) | 13 / 47 | 762 / 7544 | 2.75 (1.48, 5.10) | 0.9 | 4.67 |
| [M]SERTRALINE (Selective serotonin reuptake inhibitors (SSRI)) | 12 / 48 | 226 / 8080 | 8.94 (4.66, 17.12) | 0.9 | 8.44 |
| [D]Age 16-18 | 33 / 27 | 2659 / 5647 | 2.59 (1.55, 4.31) | 0.8 | 2.88 |
| [I]F41 (Other anxiety disorders) | 15 / 45 | 737 / 7569 | 3.42 (1.90, 6.17) | 0.8 | 7.88 |
| [M]ARIPIPRAZOLE (Antipsychotic medication) | 12 / 48 | 246 / 8060 | 8.17 (4.31, 15.64) | 0.6 | 7.17 |
| [I]Z86 (Personal history of certain other diseases) | 11 / 49 | 571 / 7735 | 3.03 (1.57, 5.87) | 0.6 | 10.5 |
| [M]ZOLOFT (Antidepressant) | 5 / 55 | 39 / 8267 | 19.30 (7.32, 50.91) | 0.5 | 5.8 |
| [I]F32 (Major depressive disorder, single episode) | 28 / 32 | 615 / 7691 | 10.91 (6.55, 18.36) | 0.5 | 8 |
| [M]ESCITALOPRAM (Selective serotonin reuptake inhibitors (SSRI)) | 10 / 50 | 115 / 8191 | 14.30 (7.03, 28.79) | 0.5 | 9.4 |
| [M]CHOLECALCIFEROL (Vitamin) | 10 / 50 | 375 / 7931 | 3.74 (1.82, 7.61) | 0.4 | 7 |
| [M]FLUOXETINE (Selective serotonin reuptake inhibitors (SSRI)) | 10 / 50 | 227 / 8079 | 7.10 (3.56, 14.15) | 0.4 | 10.75 |
| [M]QUETIAPINE (Atypical antipsychotic) | 10 / 50 | 136 / 8170 | 10.59 (5.10, 21.98) | 0.3 | 5.67 |
| [T]MDMA (ECSTASY), URINE{U} (Drug tests) | 17 / 43 | 282 / 8024 | 11.25 (6.36, 19.89) | 0.3 | 13.67 |
| [T]BILIRUBIN, DIRECT{U} (Routine tests) | 13 / 47 | 832 / 7474 | 2.48 (1.34, 4.62) | 0.2 | 6 |
| [T]TRICYCLIC SCREEN, URINE{U} (Drug tests) | 21 / 39 | 417 / 7889 | 10.18 (5.93, 17.46) | 0.2 | 8 |
| [M]GUANFACINE (Treatment of ADHD and high blood pressure) | 6 / 54 | 244 / 8062 | 3.67 (1.57, 8.58) | 0.2 | 9 |
| [I]F43 (Reaction to severe stress, and adjustment disorders) | 15 / 45 | 408 / 7898 | 6.42 (3.56, 11.70) | 0.2 | 10 |

**Table 12. Details of selected predictors.** [I] denotes ICD diagnosis code; [D] denotes demographics; [M] denotes medication; [T] denotes lab test result. For lab test results, U=Unspecified; H=High; L=Low; A=Abnormal.

| **Predictors** | **Category** | **Is female specific factor?** |
| --- | --- | --- |
| [D]Age 10-12 | Demographics | N |
| [I]R45 (Symptoms and signs involving emotional state) | Other mental health | N |
| [I]F32 (Major depressive disorder, single episode) | Depression | N |
| [D]Female Sex | Demographics | Y |
| [M]SERTRALINE (Selective serotonin reuptake inhibitors (SSRI)) | Depression | N |
| [M]HYDROXYZINE PAMOATE (Treatment of itchiness, anxiety, and nausea) | Other mental health | N |
| [T]CULTURE, URINE{U} (Routine tests) | Routine tests | N |
| [M]ETHINYL ESTRADIOL (Birth control pills) | Pregnancy | Y |
| [T]ACETAMINOPHEN LEVEL{U} (Drug tests) | Drug tests | N |
| [T]URINALYSIS WITH MICROSCOPIC{H} (Routine tests) | Routine tests | N |
| [T]POCT PREGNANCY, URINE{U} (Pregnancy) | Pregnancy | Y |
| [I]F90 (Attention-deficit hyperactivity disorders) | Other mental health | N |
| [M]LORAZEPAM (Benzodiazepine and anticonvulsant medication) | Other mental health | N |
| [T]MDMA (ECSTASY), URINE{U} (Drug tests) | Drug tests | N |
| [I]F91 (Conduct disorders) | Other mental health | N |
| [M]ESCITALOPRAM (Selective serotonin reuptake inhibitors (SSRI)) | Depression | N |
| [M]CITALOPRAM (Selective serotonin reuptake inhibitors (SSRI)) | Depression | N |
| [M]FLUOXETINE (Selective serotonin reuptake inhibitors (SSRI)) | Depression | N |
| [T]POCT URINALYSIS DIPSTICK{A} (Routine tests) | Routine tests | N |
| [M]HYDROXYZINE HCL (Antihistamine) | Other mental health | N |
| [T]URINALYSIS WITH MICROSCOPIC{U} (Routine tests) | Routine tests | N |
| [I]F31 (Bipolar disorder) | Other mental health | N |
| [I]F41 (Other anxiety disorders) | Other mental health | N |
| [I]Z86 (Personal history of certain other diseases) | Other | N |
| [M]CHOLECALCIFEROL (Vitamin) | Other | N |
| [M]VENLAFAXINE (Major depressive disorder) | Depression | N |
| [M]GUANFACINE (Treatment of ADHD and high blood pressure) | Other mental health | N |
| [I]F39 (Unspecified mood [affective] disorder) | Other mental health | N |
| [M]TRAZODONE (Serotonin modulator) | Depression | N |
| [T]POCT, PREGNANCY, URINE(ED&IP){U} (Pregnancy) | Pregnancy | Y |
| [T]SALICYLATE LEVEL{L} (Drug tests) | Drug tests | N |
| [I]M25 (Other joint disorder, not elsewhere classified) | Other | N |
| [I]Z72 (Problems related to lifestyle) | Other | N |
| [M]PROZAC (Antidepressant) | Depression | N |
| [M]RISPERIDONE (Atypical antipsychotics) | Other mental health | N |
| [M]ZOLOFT (Antidepressant) | Depression | N |
| [M]ARIPIPRAZOLE (Antipsychotic medication) | Other mental health | N |
| [T]GC/CHLAMYDIA{U} (Routine tests) | Routine tests | N |
| [D]F63 (Impulse disorders) | Other mental health | N |
| [M]QUETIAPINE (Atypical antipsychotic) | Other mental health | N |
| [T]OXYCODONE, URINE{U} (Drug tests) | Drug tests | N |

**Table 13. Model improvements across prediction windows.** Model Improvement is defined as the ratio of PPV to suicide base rate. **Abbreviations**: PPV indicates positive predictive value.

| **Prediction window** | **No. Positive** | **No. Negative** | **Base rate (%)** | **PPV** | | **Model Improvement** | |
| --- | --- | --- | --- | --- | --- | --- | --- |
|  |  |  |  | 90% Specificity | 95% Specificity | Under 90% Specificity | Under 95% Specificity |
| 0 day | 180 | 41,541 | 0.43 | 0.03 | 0.04 | 6.9 | 9.2 |
| 7 days | 177 | 19,351 | 0.91 | 0.06 | 0.08 | 6.6 | 8.8 |
| 14 days | 175 | 18,844 | 0.93 | 0.06 | 0.08 | 6.5 | 8.6 |
| 30 days | 167 | 17,620 | 0.95 | 0.05 | 0.09 | 5.3 | 9.5 |
| 60 days | 149 | 16,002 | 0.93 | 0.06 | 0.07 | 6.5 | 7.5 |
| 90 days | 139 | 14,925 | 0.93 | 0.06 | 0.07 | 6.5 | 7.5 |
| 180 days | 114 | 12,452 | 0.92 | 0.05 | 0.08 | 5.4 | 8.7 |
| 270 days | 83 | 10,337 | 0.80 | 0.05 | 0.08 | 6.3 | 10.0 |
| 365 days | 60 | 8,306 | 0.72 | 0.04 | 0.05 | 5.5 | 6.9 |

**Figure 1.** **Illustration of prediction window.** Prediction window is defined as a specific time window before the first diagnosis of suicide attempt of a positive subject, or the record of the last visit of a negative subject, in timeline. The model for each prediction window only uses data equal to or more distant in time than the length of the window. We constructed models for prediction windows including 0, 7, 14, 30, 60, 90, 180, 270, and 365 days.

**Figure 2.** **Illustration of the study flow.** Data were censored by prediction windows. Predictor screening was applied to obtain a set of candidate predictors. Data were randomly split into training and testing sets. Over the training set, predictive model was trained where a sequential forward selection approach was utilized. Model was evaluated upon testing set. We repeated the procedure 10 times to evaluate robustness of the predictive models.

**Figure 3. Receiver operating characteristic (ROC) curves for the predictive model over prediction windows.** The ROC curves illustrate the overall predictive performance of the model across test subjects.

**Figure 4.** **AUC curve cross prediction windows.** This figure plots prediction performances of the proposed Sequential selection model and Logistic Regression with Lasso in terms of AUC with 95% confidence interval cross all prediction windows. An AUC of 0.5 is the worst performance that means random guessing.

**Figure 5. Percent of patients with specific risk factor.** X-axis denotes prediction window, while Y-axis denotes percent of patients with specific risk factor category.
